# Diverse Roles of Prostaglandins in Blastocyst Implantation

**DOI:** 10.1155/2014/968141

**Published:** 2014-01-27

**Authors:** Naguib Salleh

**Affiliations:** Department of Physiology, Faculty of Medicine, University of Malaya, Lembah Pantai, 50603 Kuala Lumpur, Malaysia

## Abstract

Prostaglandins (PGs), derivatives of arachidonic acid, play an indispensable role in embryo implantation. PGs have been reported to participate in the increase in vascular permeability, stromal decidualization, blastocyst growth and development, leukocyte recruitment, embryo transport, trophoblast invasion, and extracellular matrix remodeling during implantation. Deranged PGs syntheses and actions will result in implantation failure. This review summarizes up-to-date literatures on the role of PGs in blastocyst implantation which could provide a broad perspective to guide further research in this field.

## 1. Introduction

PGs are produced from arachidonic acid which is released from the membrane phospholipids via the action of phospholipase A_2_ enzyme [1]. Arachidonic acid is converted into PGH_2_ by PG-endoperoxide synthases (PTGS), also known as cyclooxygenase (COX). COX is the key enzyme in PG biosynthesis, acting both as dioxygenase and peroxidase [2]. There are currently two identified PTGS isozymes: a constitutive PTGS1 (COX-1) and inducible PTGS2 (COX-2), which differ in their expression and tissue distribution [3]. COX dimer can be found both in the endoplasmic reticulum and nuclear membrane [2].

PGH_2_ is an unstable intermediate which undergoes rapid conversion into various other prostanoids by specific terminal PG synthases. The latter include PGE synthase (PGES), PGIS, PGDS, PGFS, and thromboxane synthase (TXS) that form PGE_2_, PGI_2_, PGD_2_, PGF_2*α*_, and TXA_2_ from PGH_2_, respectively [4]. PGES consists of microsomal (m)PGES-1, mPGES-2, and cytosolic (c)PGES [5]. Meanwhile, alpha ketoreductase (AKR)1A1 and AKR1BI have been identified as functional PGFS in humans [6]. PGI_2_, also known as prostacyclin, is non-enzymatically metabolized to a more stable form, 6-keto PGF_1*α*_ [7]. PGF_2_ can be synthesized directly from PGH_2_ or indirectly via PGE_2_ while PGI_2_ could derive either directly from PGH_2_ or indirectly via PGD_2_ [8].

PGs act via binding to its various G-protein coupled receptor (GPCR), which include four subtypes of PGE receptor (EP) (EP1, EP2, EP3, and EP4) [9], PGF receptor (FP), PGI_2_ receptor (IP), and PGD_2_ receptor (DP) which consist of DP1 and DP2 [10]. Additionally, PGI_2_ may act through a nuclear peroxisome proliferator-activated receptor-*δ* (PPAR-*δ*) [11], a ligand activated nuclear receptor. PGE_2_ has recently been reported to interact with PPAR-*δ* [12]. The binding of PGs to its specific receptor will activate series of intracellular signaling cascade. Activation of EP1 is coupled to Ca^2+^ mobilization and EP2 and EP4 trigger while EP3 inhibits adenylyl cyclase [13]. Meanwhile, FP activation is coupled to phospholipase C-inositol trisphosphate (IP_3_) pathway and Ca^2+^ mobilization [14].

PGs play an indispensable role in embryo implantation. The expression of COX-1 and -2 [15], cPGES [16], mPGES-1 and -2 [17], and prostacyclin synthase [18] has been reported at the implantation site in mice, rats, and humans. PGE_2_S and PGF_2*α*_S have been localized in the human endometrial epithelia with an increase in PGE_2_ and PGF_2*α*_ concentration in the uterine fluid during the implantationwindow period [19]. Meanwhile, COX-1 is expressed in the luminal and glandular epithelia while COX-2 is expressed in the luminal epithelia and perivascular cells during the implantation window period in humans [20]. Female mice lacking COX-2 were infertile with specific fertilization, implantation, and decidualization defects [21]. COX-1-deficient female mice were fertile; however, they develop specific parturition defect [21]. Mice deficient of PG receptor specifically EP2 have been reported to exhibit impaired reproductive functions [22]. In rodents, PGE_2_ and PGF_2*α*_ have been reported to play important role in blastocyst spacing, implantation, and decidualization while PGI_2_ (prostacyclin) has been implicated in implantation and decidualization involving PPAR-*δ* [23]. PGE_2_ and PGF_2*α*_ have also been reported to be involved in rodents' myometrial circular muscle contraction which facilitates embryo transport and spacing [24].

The synthesis of PGs and its biosynthetic enzymes in the female reproductive tract can be regulated by hormones and paracrine factors. In humans, COX-1 expression in the glandular epithelia and COX-2 expression in the luminal epithelia were significantly decreased following treatment with mifepristone, a progesterone receptor antagonist, indicating that progesterone could influence COX expression [20]. In the early pregnancy in mice, uterine COX-1 gene could be regulated by the ovarian steroids, while COX-2 gene could be regulated by the implanting blastocyst [25]. Several other hormones and cytokines have also been reported to be involved in PG synthesis. Chorionic gonadotrophin (CG) was found to regulate PGE_2_ production by human and primate endometrial epithelia [26]. IL-1 *α* was reported to induce PGE_2_ and PGF_2*α*_ secretion by the mouse uterine stromal cells *in vitro* [27]. Lysophosphatidic acid (LPA), a bioactive lipid derivative, was reported to enhance PGE_2_ synthesis and COX-2 expression in the rat uterus [28]. Activation of epithelial Na^+^ channel (ENaC) in the mouse endometrial epithelium by embryo-released serine protease, trypsin, has recently been reported to trigger Ca^2+^ influx that could lead to PGE_2_ release, CREB transcription factor phosphorylation, and upregulation of COX-2 enzyme [29].

An understanding on the role of PG in blastocyst implantation is far from complete. In view of this, we aim to summarize literatures related to PG role in implantation particularly in humans and rodents in order to provide a broad perspective to guide further research in this field.

## 2. Role of PGs in Increased Vascular Permeability and Angiogenesis at the Implantation Site

Increased vascular permeability and stromal edema are two of the earliest signs following blastocyst attachment [30]. In mice, increased vascular permeability could be seen at day 4.5 of the oestrous cycle, as evidence from a contrast-enhanced (CE)-MRI and fluorescence microscopic studies [31]. Changes in vascular permeability are followed by progressive increase in angiogenesis [31]. Sex steroids have been reported to exert differential effect on these changes with estrogen increases the permeability but profoundly inhibits angiogenesis *in vivo*, while progesterone stimulates angiogenesis however has little effect on permeability [32].

Changes in vascular permeability and angiogenesis at the time of embryo implantation were caused by differential expression of proangiogenic factor in the uterus which include the vascular endothelial growth factor (VEGF) and its receptors [33]. VEGF together with angiopoietin (Ang)-1 and Ang-2 direct angiogenesis during decidualization. Ang-1 in collaboration with VEGF induces vessel maturation and maintains vessel leakiness, whereas Ang-2 induces vessel destabilization required for further sprouting [34]. The expression of Ang-like 4 gene has been reported in the uterus at the time of decidualization in response to PPAR agonist [35] while angiomotin (Amot-2), a vascular angiogenesis-related protein, has recently been reported to be expressed in the endometrial stroma under the progesterone influence [36].

PGs and platelet-activating factor (PAF) are important paracrine factors involved in the increase in vascular permeability at site of embryo implantation [37]. PAF receptor (PAF-R) mRNA was detected in the endometrial glands during the secretory phase of the menstrual cycle [37]. Interaction between PAF and its receptor resulted in a rapid release of nitric oxide (NO), a potent vasodilator, increased VEGF expression, and activates focal adhesion kinase, FAKpp125 [38]. PAF-evoked NO release was dependent on protein kinase C (PKC) and extracellular Ca^2+^ [39].

PGE_2_ was found to be more effective than prostacyclin (PGI_2_), PPAR-*δ*, and retinoic acid (RXRA) in causing an increase in endometrial vascular permeability in rats [40]. PGE_2_ mediates sex-steroid effect on VEGF and angiopoietin [34] expression which resulted in increased vascular permeability and angiogenesis during implantation and decidualization, respectively [41]. In contrast, the activity of nitric oxide synthase (NOS), an enzyme responsible for NO production which was reported to be the highest at the site of embryo implantation [42], was inhibited by PGE_2_ [43], suggesting that PGE_2_ could also be involved in the control of the extent of vascular permeability induced by NO. On the other hand, inducible NO itself has been reported to affect COX-2 activity and thus could affect the PG synthesis [44].

The involvement of PGF_2*α*_ in NO synthesis and blood flow to the implantation site is relatively unknown; however, PGF_2*α*_ was found to cause acute increase in blood flow to the corpus luteum by stimulating the activity of epithelial nitric oxide synthase (eNOS) [45]. Additionally, PGF_2*α*_ was also found to affect angiogenesis as reported in the endometrial adenocarcinoma tissue [46]. Prostacyclin (PGI_2_), a potent vasodilator, has been reported to play important role in the increase in vascular permeability at the implantation site. The level of prostacyclin increases in early pregnancy and is the main eicosanoid produced by the endothelia of the smooth muscle arteries in parallel with the increase in the expression of PGIS [47]. Prostacyclin binds to IP in the glandular epithelial cells, resulting in rapid activation of extracellular signal regulated kinase (ERK)1/2 as well as inducing the expression of proangiogenic genes, basic fibroblast growth factor (bFGF), and Ang-1 and -2, via cross talk with the epidermal growth factor receptor (EGF-R) [48].

## 3. Role of PGs in Decidualization

Decidualization is the most important event attributed to PGs, which is defined as differentiation of the elongated stromal fibroblasts into secretory, epithelioid-like decidual cells. In rodents, this process is initiated by the implanting blastocyst [34] while in humans decidualization begins immediately following ovulation, reaches the peak in the mid-luteal phase of the menstrual cycle [49], in response to progesterone, and is independent of the blastocyst signal [50]. Transformation of the stromal fibroblasts into decidual cells can first be seen in the vicinity of the terminal spiral arteries which then spread throughout the endometrial compartment. The decidualized stromal cells immediately surrounding the implanting blastocyst cease proliferating and formed a primary decidual zone (PDZ) [51, 52]. Cells surrounding PDZ continue to proliferate and differentiate into polyploid decidual cells which formed secondary decidual zone (SDZ) [34]. Decidualization is associated with polyploidy, that is, formation of multinucleated (mono- and binucleated) and giant cells [53, 54] due to the altered expression and functional activity of the cell cycle regulatory molecules [55] such as cyclin D_3_, which can be induced by heparin-binding epidermal growth factor (HB-EGF) [54].

Decidualization is characterized by enhanced production of insulin-like growth factor-binding protein-1 (IGFBP-1), prolactin (dPRL), and forkhead transcriptional factor (FOXO1) in response to hormonal stimulation [49, 56]. The expression of decidual specific genes that encode these proteins requires cAMP [57], progesterone [58], and the recently identified ERK1/2 [59] signaling. The binding of peptide hormones and prostanoids to GPCR will result in the activation of adenylate cyclase, an enzyme involved in cAMP synthesis [60]. cAMP will then phosphorylate protein kinase A (PKA), which consist of regulatory and catalytic subunits [61]. Binding of cAMP to the regulatory subunit will result in the release and activation of the catalytic subunit which will phosphorylate target molecules in the cytoplasm or transcription factors in the nucleus [62]. Persistent rise in intracellular cAMP level is required to maintain the decidualized phenotype [63] in which cAMP withdrawal will cause the decidualized stromal cells to reacquire an undifferentiated phenotype [64].

PKA phosphorylates cAMP response element binding protein (CREB) [58] and the related cAMP response element modulator (CREM) [65]. Phosphorylation will also recruit coactivator CREB binding protein (CBP) to the promoter region of the target genes [50], facilitating DNA transcription [66]. A rise in intracellular cAMP will cause Ca^2+^ to enter the cell via TRPC1 channel which is essential for the initiation of decidualization [67]. Decidualization process can be inhibited by several factors including transforming growth factor (TGF)-*β*1 [68] and Krüppel-like factor 12 transcription factor [69]. Phosphodiesterases, an enzyme that degrades cAMP into AMP which result in a decrease in cAMP level, was also found to inhibit decidualization [70].

Peptide hormones implicated in the decidualization process include relaxin [71], luteinizing hormone/human chorionic gonadotrophin (LH/hCG) [72], and corticotrophin releasing hormone (CRH) [73], while PGE_2_ is the only prostanoid involved [74]. Other factors include insulin and insulin-like growth factor (IGF-I and II) [75], transforming growth factor (TGF)-*β* [76], epidermal growth factor (EGF), and platelet-derived growth factor (PDGF) [77]. PGE_2_ has been reported to cause an increase in intracellular cAMP level and stimulates the activity of alkaline phosphatase (ALP) [78] via EP2 and EP4 receptors [79]. Meanwhile, PGE_2_ and relaxin, partly via cAMP/PKA-dependent pathway, have been reported to stimulate IL-11 secretion which cause a direct increase in the cAMP level [80].

Decidual cells have been reported to synthesize and secrete PGs [81] and express PG receptors [82]. PGs can also be transported into these cells via the prostaglandin transporter (PGT). Evidences for the increase in PG synthesis include upregulation of COX-1, COX-2, cPGES, and mPGES expression in mice [16, 83], rats [18, 84], guinea pigs [85], and humans [86, 87] and upregulation of AKR1B1, a highly functioning PGF synthase responsible for PGF_2*α*_ production in humans [88]. In early pregnancy in rats, COX-2 expression is increased between days 2 to 5 [89] suggesting that PGs are required in the process of stromal cells decidualization. While PGE_2_ and PGF_2*α*_ are the main PGs involved, prostacyclin (PGI_2_) has also been implicated in decidualization in view that in mice, the expression of PGIS significantly increases at day 5 and gradually decreases thereafter [90]. In addition to the elevated expression of PG biosynthetic enzymes, the reported increase in the expression of EP2 and PPAR-*δ* in mouse decidual cells further provides evidence on the involvement of PGs in this process [91].

Progesterone is essential for decidualization in both humans and rodents. Progesterone exerts its effect via binding to the nuclear progesterone receptor-A (PR-A), which interacts with the transcription factors including CCAAT/enhancer-binding protein *β* (C/EBP*β*) [92], forkhead proteins [93], and signal transducer and activator of transcription 5 (STAT5) [94]. Progesterone receptor (PR)-interacting protein Krüppel-like factor (KLF) 9, which expression is high in the predecidual stroma, interacts with bone morphogenetic protein 2 (BMP2) to maintain stromal cells sensitivity towards progesterone [95]. C/EBP*β* is also essential for the cAMP signaling [96]. C/EBP*β* mediates activation of the decidual PRL promoter, resulting in the transcription of dPRL gene [50]. In humans, a functional link between C/EBP*β* and STAT3 was found to be crucial in the regulation of endometrial stromal cell differentiation [97].

In addition to dPRL, PR also participates in the transcriptional regulation of IGFBP-1 gene [98]. PR could be involved in regulating Snail, a transcription repressor which has recently been identified to play a central role in the epithelial-mesenchymal transition in which its expression has been reported to be induced by HB-EGF via EGFR-ERK-STAT3 signaling pathway [99]. HOXA-10, an abdominal-like homeobox gene reported to be involved in the decidualization process, could also be influenced by PR where loss of Hoxa-10 function in mice has been reported to result in infertility [100].

The involvement of PGs in the progesterone-induced decidualization has been documented. COX-2 was reported to regulate the expression of Snail transcription repressor [99]. Dysregulation of EGF and COX-2 expression in the mouse uteri during peri-implantation period which is associated with high plasma progesterone level resulted in implantation failure [101]. Meanwhile, progesterone was also found to upregulate the expression of EP2 [102], while progesterone and HOXA-10 have been reported to upregulate the expression of EP3 and EP4 in the stroma [100]. Apart from this, other roles of PGs in these processes remain unknown.

## 4. Role of PGs in Extracellular Matrix Remodeling 

The extracellular matrix (ECM) is composed of collagens, noncollagenous multiadhesive glycoproteins, elastin, hyaluronan, proteoglycans, and glycosaminoglycans [103]. In early pregnancy, uterine ECM plays important role in decidualization, embryo attachment, trophoblast invasion, and maintenance of pregnancy [104]. ECM has been reported to undergo extensive remodeling in preparation for blastocyst adhesion, trophoblast invasion, and placentation. Changes in ECM composition are characterized by phagocytosis and enzymatic digestion of collagen fibrils, increased collagen fibril diameter, deposition of basement membrane proteins, synthesis and secretion of sulfated glycosaminoglycans, and decrease in the number of elastic fibrils surrounding the matured decidual cells [105].

Sex steroids, cytokines, PGs, and growth factors have been reported to affect endometrial ECM composition in early pregnancy [106]. Dynamic changes in ECM are evidenced from the spatiotemporal changes in the expression of matrix metalloproteinase enzyme (MMP) and tissue inhibitor of metalloproteinase (TIMP) isoforms throughout the estrus cycle and in early pregnancy. These two enzymes participate in the ECM degradation and remodelling. The expression of MMP-2 mRNA was observed in the stroma between days 3 and 5 and in the secondary decidual zone on day 6, while MMP-9 mRNA was expressed in the trophoblast giant cells on day 8 of pregnancy in mice [107]. Meanwhile, the expression of TIMP-1, 2, and 3 was detected in the primary decidual zone on days 2 to 5 and in the primary and secondary decidual zone on days 6 to 8 of early pregnancy in mice [107] and rats [108, 109]. TIMPs have been proposed to regulate the extent of trophoblast invasion [110]. Human endometrial stromal cells secrete TIMP-3 which play essential role in early implantation by modulating the trophoblast invasion [111].

Progesterone [112, 113], 17*β*-estradiol [114], urokinase-plasminogen activator [115], leukaemia inhibitory factor (LIF) [116], tumour necrosis factor (TNF) and interferon-*γ* [117], transforming growth factor beta (TGF*β*), interleukin-1 and interleukin-6 (IL-1, IL-6) [118], lipopolysaccharides [119], epidermal growth factor (EGF) [120], insulin-like growth factor (IGF), and insulin-like growth factor binding protein-1 (IGFBP-1) [121] as well as trophoblast factors including hCG [122] have been reported to affect the expression and activities of MMPs and TIMPs. The effect of PGs on uterine ECM degradation and remodeling is however poorly understood. Limited observations suggested that PGF_2*α*_ might be involved in ECM turnover via affecting the expression of MMP2, cathepsin L, TIMP2 and TIMP3, plasminogen activator inhibitor1 (PAI1), tissue type plasminogen activator (tPA), urokinase plasminogen activator (uPA), endothelin 1, calponin, carboxypeptidase D and calponin acid [123]. Meanwhile, in the cervix, PGE_2_ via EP2 and EP4 has been reported to stimulate hyaluronan synthesis in the remodeling of cervical ECM [124] while PGF_2*α*_ and IL-1_*α*_ have been reported to stimulate the secretion of MMP-1 which plays important role in the degradation of extracellular collagen types I and III [125].

## 5. Role of PGs in Leukocyte Infiltration 

A profound influx of uterine natural killer (NK) cells and macrophages is essential for successful implantation [126, 127]. In the murine deciduas, NK cells are progressively inactivated by PGE_2_ produced by the decidual cells and decidual macrophages [128]. Meanwhile, PGE_2_ secretion by the first trimester human decidua blocks activation of the maternal decidual leukocytes with a potential antitrophoblast killer function by inhibiting *in situ* IL-2 receptor generation and IL-2 production [129]. PGF_2*α*_ may be involved in the inflammatory response by regulating neutrophil chemotaxis as reported in the endometrial adenocarcinoma tissue [130].

## 6. Role of PGs in Embryo Transport 

The transport of gametes and embryos which involved both muscular contraction and ciliary activity is an important function of the Fallopian tube [131]. Progesterone is required for the normal embryo transport along the oviduct [132, 133]. PGs, a known mediator of muscular contractility, has long been documented to be involved in the oviductal embryo transport [134]. PGs mediate both contraction [135] and relaxation [136] of the smooth muscle. Epithelial-derived PGs activate DP, EP2, EP4, and IP receptors which cause an increase in intracellular cAMP level [cAMP]_i_, resulting in smooth muscle relaxation [137]. On the other hand, EP1 and FP activation which coupled to Ca^2+^ mobilization resulted in the smooth muscle contraction [138].

Different EP isoforms that have been identified along the female reproductive tract, with their activation, can either cause increased or decreased intracellular cAMP (cAMP)_i_ or increased intracellular Ca^2+^ level though usually resulting in the smooth muscle contraction [139]. The expression of EP and FP has been reported in the human Fallopian tubes as evidence from the increased smooth muscle contraction following treatment with PGF_2*α*_ and PGE_2_ [140]. COX-2, PGIS, and IP receptor have also been reported to be expressed in human Fallopian tubes [141] which could serve as autocrine regulator for the oviductal smooth muscle contraction [142].

## 7. Role of PGs in Blastocyst Growth and Development

Coordinated growth and development of the embryos from 2- to 4- and subsequently 8-cell into morula and blastocyst stages is a prerequisite for successful implantation [143]. During development, blastocyst expresses multiple factors and their receptors in response to sex steroids and growth factors, which in turn regulate blastocyst growth and participate in the signal exchange with the receptive endometrium. Hormones and factors expressed include the preimplantation factor (PIF) [144], chorionic gonadotrophins (cG) [145], leukemic inhibitory factor (LIF) [146], heparin-binding epidermal growth factor (HB-EGF) [147], and PGs [148].

Prostacyclin (PGI_2_) is the most abundant PGs produced by the mouse blastocysts. In addition, the 8-cell, morula, and blastocyst stages also synthesize PGE_2_ [149]. PGI_2_ binds to IP receptor and is involved in regulating embryo development [150]. Meanwhile, COX-1, COX-2, and PGIS have also been reported to be expressed in 4-cell stage embryos and beyond and in the inner cell mass and trophectoderm of the mouse blastocysts [151]. In the golden hamsters, COX-2 expression in 8-cell stage embryos through the hatched blastocysts was localized mainly in the blastocysts' trophectoderm was critical for blastocyst hatching [152]. PGI_2_ has also been reported to regulate blastocyst cells apoptosis by acting as an antiapoptotic factor [149]. Meanwhile, EP2 and FP expression which have been detected in mouse blastocysts' trophectoderm and inner cell mass participated in embryo adhesion [19].

In addition to PGI_2_, PGE_2_ also plays important role in embryo development [148]. mPGES mRNA was detected at all stages during preimplantation embryo development [83] while cPGES expression has been reported in 2-cell, 4-cell, and 8-cell, morula, and blastocyst stages [16] in mice. Preimplantation mouse embryos also express PPAR-*δ*, which is essential for enhancing the PGI_2_ effect on blastocyst hatching where impaired blastocyst formation and hatching have been reported in the PPAR-*δ* deficient embryos [153].

## 8. Role of PGs in Trophoblast Invasion

Following blastocyst adhesion, trophoblast cells differentiate and acquire the invasive phenotype [154]. The role of PGs in facilitating trophoblast invasion is largely unknown; however, several evidences indicate its involvement in this process. PGE_2_ and EP2 agonist have been reported to increase the adhesiveness of human HTR-8/SVneo trophoblast cell line (human trophoblast-derived cell line) to the ECM via MEK/MAPK signaling pathway as well as upregulating the expression of cell adhesion protein such as focal adhesion kinase and intercellular adhesion molecule such as integrins [155]. Additionally, the expression of EP2 has also been reported in the trophoblast which could be stimulated by PGE_2_ via autocrine signaling [155].

The expression of COX-2 and PGE_2_ synthase was also detected in the human HTR-8/SVneo cells [156]. Co-stimulation by LIF and IL-1*β* induced higher amount of PGE_2_ production and further migration of these cells [157]. The decidua-derived factors including PGs have been reported to increase trophoblast cell invasiveness by reducing TIMP1 and TIMP3, however up-regulate TIMP2 expression, increase the mRNA expression of integrin-5 and integrin-6, but not integrin-*α*V subunit, elevate the expression of MMP2, MMP3, and MMP9 mRNA and increase the activity of MMP2 and MMP9 [158]. Meanwhile, a contradicting report indicated that PGE_2_ inhibits extravillous trophoblast cell functions, which could help to prevent excessive trophoblast proliferation and migration [159].

## 9. Conclusion

The understanding on the role of PG in blastocyst implantation and early pregnancy is far from complete. While most information was obtained from animal studies especially in rodents, more researches need to be performed in humans in order to further explore the mechanisms underlying the diverse PGs action on multiple processes of implantation.
